# Previous exposure to traumatic brain injury alters acute stress responses in mice

**DOI:** 10.1038/s41598-025-30105-2

**Published:** 2025-11-26

**Authors:** Hao Gu, Johanna Christina Reiners, Daniela Sinske, Stefan O. Reber, Bernd Knöll

**Affiliations:** 1 Institute of Neurobiochemistry , Ulm University Albert-Einstein-Allee , 11, 89081 Ulm, Germany; 2 Department of Psychosomatic Medicine and Psychotherapy Laboratory for Molecular Psychosomatics, Ulm University Medical Center Albert-Einstein-Allee, 11, 89081 Ulm, Germany

**Keywords:** Acute stress, Traumatic brain injury, Hypothalamus-pituitary-adrenal (HPA) axis, Immediate early gene, Catwalk, Corticosterone, Diseases, Neurology, Neuroscience

## Abstract

Acute stress (AS) exposure and traumatic brain injury (TBI) have a high prevalence and result in emotional and behavioral impairments. In fact, TBI is a leading cause of mortality in young adults and survivors cope with neuropsychological sequelae including memory decline, addiction, anxiety and compromised stress responses. So far, the interaction of psychological stress with previous TBI is poorly understood. Herein, the impact of a previous TBI on AS responsiveness was investigated. For this, a model of mild TBI and restraint AS was employed and mice were grouped into sham, TBI alone, AS alone and TBI + AS cohorts. The impact of an initial TBI on subsequent AS was analyzed by immediate early gene (IEG) and neuroinflammatory gene expression as well as proteomics. In addition, behavioral consequences were monitored by open field, ladder walk and the Catwalk gait analysis. Finally, since both TBI and AS impinge on hypothalamus-pituitary-adrenal (HPA) axis regulation, CORT and ACTH levels were measured. We observed that some AS associated responses such as gene induction in the brain and HPA axis were largely unaffected by TBI occurring one day before AS exposure. In contrast, on the behavioral level, several AS associated locomotor alterations were modulated post-TBI. Hormone levels of the HPA axis were likewise modulated by the TBI and AS interaction. Finally, blood-derived proteomics identified proteins regulated by individual exposures as well as by the TBI and AS interaction. In summary, we demonstrate that previous TBI affects responsiveness to AS exposure on behavioral level and HPA axis regulation.

**Supplementary Information:**

The online version contains supplementary material available at 10.1038/s41598-025-30105-2.

## Introduction

Traumatic brain injury (TBI) is one of the leading causes of disability and death in young adults^1^. In the majority of cases worldwide, TBI is considered mild (mTBI; Glasgow Coma Scale score of 13–15^2,3^; and patients typically survive. However, following TBI, patients frequently present neuropsychiatric problems such as depression, anxiety, posttraumatic stress disorder (PTSD) and substance abuse^4,5^. For instance, patients with mTBI had a significantly higher rate of major depressive disorder compared to other trauma patients at three months (reviewed in^6^. In addition, veterans with TBI reported more severe depression symptoms than those without TBI, and these depression symptoms seem to be exacerbated upon the introduction of stress^7^. Also, the probability to develop PTSD is almost threefold enhanced by TBI compared to control patients without TBI^8^. The aforementioned data suggest that in patients, TBI changes the responsiveness and resilience of towards coping with daily stress challenges.

In current research, such interactions between an initial TBI and subsequent psychological stress in patients are just beginning to be addressed^9–11^. On the molecular and cellular level, one mechanism by which physical and psychological stressors might converge is through modulation of the hypothalamus-pituitary-adrenal (HPA) axis. Psychological stress results in adrenocorticotropic hormone (ACTH) and cortisol (corticosterone, CORT in mice) release from the pituitary and adrenal cortex, respectively. In contrast, TBI patients encounter HPA axis dysfunction and may develop hypopituitarism^12–15^.

In recent years, animal models helped to disentangle the intricate relationship between TBI and stress. For instance, hyper-responsiveness to subsequent stress as observed by heightened CORT release was observed post-TBI in a rat model^16^. Furthermore, mice that received post-TBI stress had increased anxiety-like behavior compared to TBI-only groups^17^. An interaction of TBI and stress with respect to neuroinflammation was also documented by enhanced CCL2 chemokine levels in mice receiving TBI and post-injury stress^18^. Finally, on histological level, blood brain barrier (BBB) leakage was more pronounced in a rodent TBI model with post-injury restraint acute stress (AS) compared to TBI alone^19^.

So far, the interaction between an initial TBI and subsequent stress responsiveness was not analyzed in great detail with regard to consequences on behavior and gene expression. In a previous study employing the reverse order of events (AS before TBI) we showed alterations in body weight, memory function, locomotion and gene expression^20^. This encouraged us to analyze whether behavioral responsiveness and gene induction were altered in mice challenged with restraint AS post-TBI. For this, we employed the CatWalk system which allows for a detailed analysis of TBI associated gait alterations as reported before^21,22^. So far, no AS-dependent gait alterations have been reported in the literature for which we provide first data in this study. From a molecular point of view, induction of gene expression was analyzed by immediate early gene (IEG) and regeneration associated gene (RAG) expression. RAGs including *Atf3* (activating transcription factor 3) and IEGs including *cFos*,* Fosb* and *Npas4* are rapidly upregulated by various stimuli including neuronal activation as demonstrated before^20,23–25^.

We employed a weight-drop model of TBI targeting either the sensory (TBI_S_) or motor (TBI_M_) cortex followed by a single short-term (45-minute) AS exposure by restraining the mice. Our data revealed no influence by TBI on AS associated gene expression neither in the brain nor along organs of the HPA axis. In contrast, post TBI we observed a trend towards AS-associated behavioral changes such as locomotor activity and gait patterns. Finally, proteome analysis uncovered proteins regulated individually by TBI and AS, as well as by their interactions.

In summary, we identified distinct impacts of TBI on subsequent AS-associated responses in a rodent model. This indicates that even a mild TBI can interfere with the normal processing of acute stress, affecting behavioral performance, circulating biomarkers and HPA axis response.

## Materials and methods

### Mice

The study used wild-type mice (aged 14–16 weeks) of both sexes on a C57BL/6 N genetic background. All animals used in this study were obtained by in-house breeding. Mice were housed under standard laboratory conditions with a temperature of 22 °C, 60% humidity, a pathogen-free environment, and a 12-hour light/dark cycle. Food and water were provided *ad libitum*. All experimental procedures were approved by the regional authority (Regierungspräsidium Tübingen, Germany; Number: 1423) and conducted in accordance with institutional and governmental ethical guidelines. All methods were performed in accordance with the ARRIVE guidelines 2.0 for animal experiments and regulations.

**Fig. 1:**
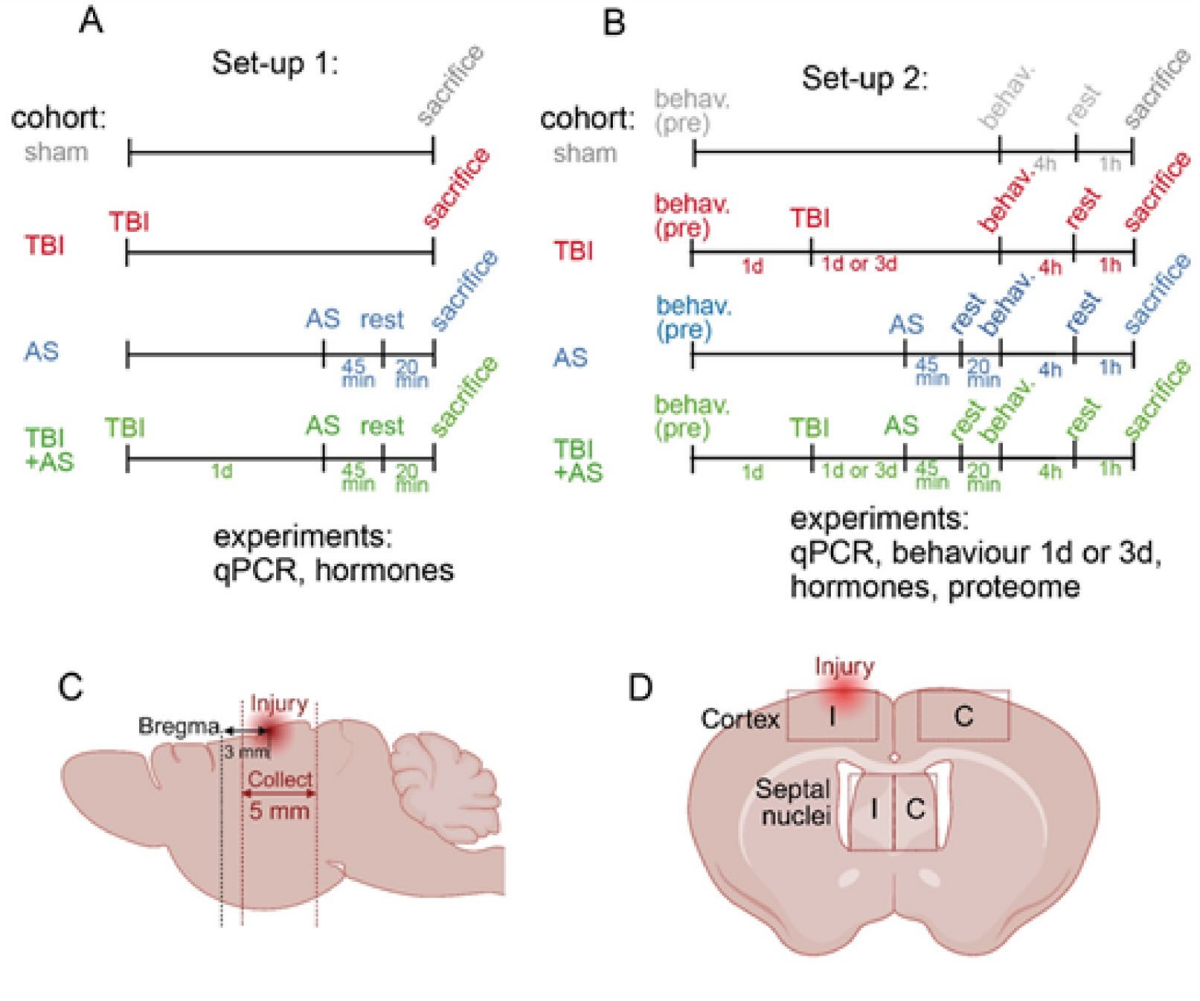
Experimental time-line and cohorts. In all experiments the four cohorts included sham (grey), TBI alone (red), AS alone (blue) and TBI preceding AS (TBI + AS, green). (**A**) In set-up 1, sham surgery or TBI was applied. 1 d later, AS was applied for 45 min or AS was omitted (for sham and TBI only). 20 min after AS was terminated, animals were sacrificed for qPCR and analysis of hormone status. (**B**) Set-up 2 was identical to set-up 1 with the exception that 20 min after AS completion, behavioral (“behav.”) analysis (OF, ladder walk, Catwalk) was performed for approx. 4 h. Animals were sacrificed 1 h after last behavior test. Proteomics was also performed with this set-up. (C, D) Schemes depicting the location of the injury site and brain areas collected for analysis in sagittal (**C**) and coronal (**D**) orientation. Abbreviations: I, ipsilateral, C, contralateral.

Mice were grouped in four cohorts according to two different experimental set-ups as outlined in Fig. 1. In the sham group, mice underwent skull exposure with a slight touch of the weight drop on the skull. In the TBI group, the same surgery was followed by a weight drop impact on the skull overlaying the cortex. In the AS group, mice received the same sham surgery as the sham group, followed by AS at one or three days post-sham surgery according to set-up 1 or 2 (Fig. 1). In the TBI + AS group, mice underwent both the TBI and AS procedures.

N-numbers were indicated by squares and circles in each figure. The n-numbers used were as follows: For set-up 1: sham (8 animals), AS (7 animals), TBI (7 animals) and TBI + AS (7 animals). For set-up 2: sham (10 animals), AS (10 animals), TBI (10 animals) and TBI + AS (10 animals). Lower numbers in individual panels were due to removal of outliers as suggested by outlier analysis with GraphPad Prism.

### TBI model

The modified weight-drop TBI model, previously reported^20^, was used to induce traumatic brain injury. A 120-gram weight was used and the falling height was set at 40–45 cm. A spacer controlled the impact depth, which was set to 2.5 mm for TBI_S_ and 2.25 mm for TBI_M_. Mice were acclimated in the operating room for 1 h, and their body weight was recorded before anesthesia. They were then placed in an anesthesia tube and inhaled a mixture of 4–5% sevoflurane and 800–900 mL/min oxygen for 2 min. Anesthesia depth was confirmed by the loss of pedal and tail withdrawal reflexes, followed by subcutaneous administration of buprenorphine (~ 0.04 mg/kg) for analgesia. The scalp was shaved, disinfected with 70% ethanol, and a 1 cm midline incision was made to expose the skull.

The mouse was transferred to the TBI apparatus and its head secured in a holding frame. The impact site was precisely positioned using the 3-axis mobile platform of the TBI apparatus. For TBI_S_, the impactor (3 mm diameter) was aligned with the bregma and adjusted 3 mm posterior and 2 mm lateral. For TBI_M_ the impactor was adjusted 0.5 mm posterior and 1.5 mm left lateral from the bregma. The weight was released to strike the impact site at the set depth provided by the spacer. After the impact, vital signs were monitored until regular breathing resumed, after which the scalp was sutured with 6.0 proline surgical thread. Mice were under anesthesia until this point. Next, mice were placed under a heating light in a recovery cage. Surgeries were performed on a heating pad set to 37 °C, lasting approximately 10 min for non-TBI groups and 15 min for TBI groups. All surgeries were conducted between 9 a.m. and 12 p.m. Post-surgery, health status and body condition scores were monitored, with buprenorphine administered before the dark phase and again 12 h later (also for all other cohorts).

### Acute stress

AS was induced in mice using a body-restraint model^20,25^. Mice were placed in a well-ventilated 50 mL Falcon tube, which was secured with tape to prevent movement, and covered with an opaque box for 45 min to induce stress. After the restraint period, mice were returned to their home cage and allowed to rest for 20 min. AS was conducted between 9 a.m. and 12 p.m.

### Behavioral tests and body weight measurement

All behavioral tests were conducted between 9 a.m. and 3 p.m., with mice allowed 1 h to acclimate before testing. The order of behavior tests was OF (open field) followed by LW (ladderwalk; 1 d between TBI and AS) or OF followed by Catwalk (3d between TBI and AS).

Behavior data obtained before (“pre”) surgical intervention served as reference for calculation of alterations recorded after individual or combined TBI/AS application (“post”) according to the following calculation (value _post_ – value _pre_/value _pre_ X 100).

#### Body weight

Body weight was measured before anesthesia and after the completion of behavioral tests.

#### OF

To assess locomotor and exploratory performance, the OF test was conducted 1 day pre-surgery and 1 or 3 days post TBI. Mice were placed at the center of a square OF arena (50 × 50 cm) and recorded for 15 min using a video camera. Locomotor activity, measured as total track length, was analyzed using Viewer III software (Biobserve, Bonn, Germany), as previously described^20,23^.

#### LW

To evaluate walking coordination, the ladder walk test was performed 1 day pre- and post-surgery. The apparatus consisted of two clear Plexiglas sidewalls (69.5 × 15 cm) connected by 8 cm metal rungs (2 mm diameter) spaced 1 cm apart, spanning a total length of 60 cm. The wall distance was adjusted to permit forward movement while preventing turning. Each mouse traversed the ladder three times spontaneously, with trials recorded using a camera. Videos were analyzed at ¼ speed to quantify slips from all limbs, and the average number of slips per run was calculated^20,23^.

#### Catwalk

To detect fine gait impairment, the Catwalk test was employed 1 day pre-surgery and 3 days post-surgery. The CatWalk XT^®^ system (version 10.6, Noldus, Wageningen, Netherlands) is an automated gait analysis platform consisting of a 1.3 m horizontal glass plate covered by a removable tunnel to provide dimmed lighting. A green LED light internally reflected within the glass plate was refracted upon paw contact, with illuminated areas captured by a high-speed color camera positioned beneath the plate. Gait parameters were automatically analyzed using CatWalk XT^®^ software. Calibration settings included a maximum speed variation of 60%, a camera gain of 20.00 dB, and a detection threshold of 0.1 a.U.

After one hour of acclimatization, each mouse was placed at the walkway’s start and voluntarily traversed toward the opposite end, where its home cage was positioned beneath. A minimum of three uninterrupted runs qualified for analysis was required. There was no time limit for per run, and identical calibration settings were applied across all mice. In most cases, three valid runs were obtained within five trials, with a maximum of ten trials permitted per mouse.

Baseline data collection represented the mice’s first exposure to the CatWalk XT^®^ system. To enhance data accuracy, automated footprint recognition was manually reviewed.

### Dissection and quantitative real-time PCR (qPCR)

Mice were euthanized in a flooded CO₂ chamber (less than 1 min) and decapitated upon loss of consciousness. Brains, pituitaries and adrenal glands were dissected, flash-frozen and stored at −80 °C. Whole blood was immediately collected after decapitation into EDTA microvettes, centrifuged (10 min, 4500 g), and the supernatant was flash-frozen and preserved at −80 °C.

After immersion in ice-cold PBS for 3 min, each brain was sectioned into 1 mm-thick coronal slices using a tissue chopper. Slices were transferred to a petri-dish with ice-cold PBS, and individual sections were separated under a microscope. Regions of interest, including the septal nuclei, hypothalamus and cortex were dissected using a surgical scalpel and tweezers and collected from the corresponding slices.

Total RNA was extracted using the RNeasy Mini Kit (Qiagen) following the manufacturer’s instructions. Reverse transcription was performed using 1 µg of RNA, reverse transcriptase (Promega), and random hexamers.

**Fig. 2:**
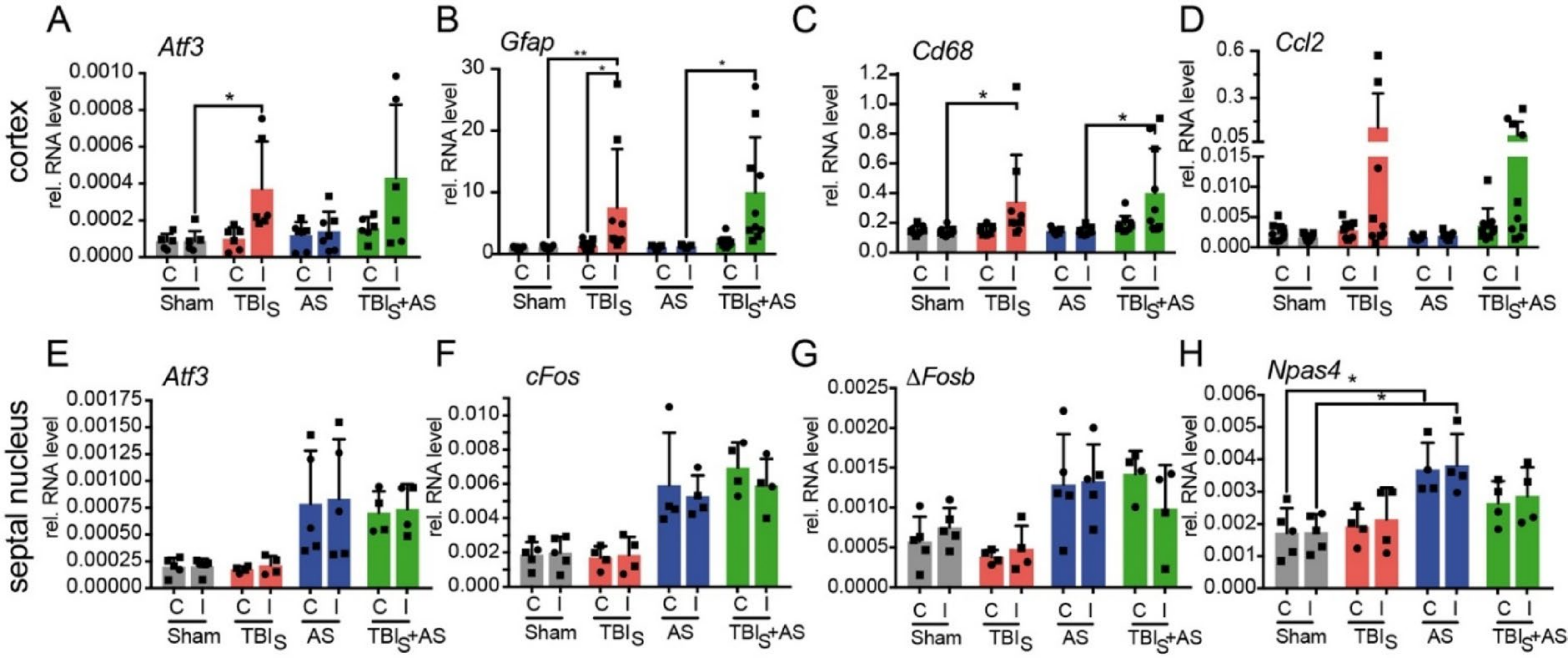
Selected gene expression responses are unaffected by TBI and AS interaction. (**A**-**D**) Cortical tissue was subjected to qPCR analysis at 1 d (A, set-up 1) or 1 d (B-D, set-up 2) after TBI. In the ipsilateral (“I”) cortex hit by the TBI impact, *Atf3* (**A**), *Gfap* (**B**), *Cd68* (**C**) and *Ccl2* (**D**) were upregulated by TBI alone whereas the contralateral (“C”) cortex was not affected. This response was almost similar in TBI + AS treated animals. (**E**-**H**) In the septal nucleus, *Atf3* (**E**) and three IEGs, *cFos* (**F**), *Fosb* (**G**) and *Npas4* (**H**) were upregulated by AS alone in both hemispheres. This AS mediated response was similar when TBI preceded AS (TBI + AS; E-H). N-numbers are indicated by individual squares and circles in each bar. Squares represent male whereas circles represent female mice.

qPCR was conducted using the Roche Light Cycler 480 (Roche), with a 10 µL reaction mixture per well, comprising 2 µL cDNA, 3 µL specific primers, and 5 µL SYBR Premix Ex Taq (Tli RNase H Plus) PCR Master Mix (TaKaRa Bio Europe, Saint-Germain-en-Laye, France). Cycle threshold values were determined using LC480 II software. Relative mRNA expression of target genes was calculated using the ΔCt method, with *Gapdh* (glyceraldehyde-3-phosphate dehydrogenase) or hypoxanthine phosphoribosyltransferase (*Hprt*; Fig. 2B, C, D) as the housekeeping gene. All experiments were performed in technical duplicates. Primer details are available upon request. Data in Fig. 2B-D were from set-up 2 (Fig. 1).

### Proteomics

For the in-solution digest, 6 µg of plasma protein was reduced with 5 mM DTT (AppliChem, Darmstadt, Germany) for 20 min at RT and subsequently alkylated with iodoacetamide (Sigma-Aldrich, St. Louis, USA) for 20 min at 37 °C. Trypsin (Thermo Scientific, Rockford, IL, USA) was added in a 1:50 enzyme-protein ratio and digested overnight at 37 °C.

Employing an LTQ Orbitrap Elite system (Thermo Fisher Scientific, Bremen, Germany) online coupled to an U3000 RSLCnano (Thermo Fisher Scientific, Idstein, Germany), samples were analyzed as described previously^26^. Samples were analyzed using an LTQ Orbitrap Elite system (Thermo Fisher Scientific, Bremen, Germany) online coupled to an U3000 RSLCnano (Thermo Fisher Scientific, Idstein, Germany), as previously described^26^. Database searches were conducted using MaxQuant Ver. 1.6.3.4^27^ with the Andromeda search engine^28^, correlating MS/MS spectra to the UniProt mouse reference proteome (www.uniprot.org). Carbamidomethylated cysteine was set as a fixed modification, while oxidation (M) and acetylated protein N-termini were considered as variable modifications. False discovery rates for peptides and proteins were set to 0.01. Statistical analysis was performed using LFQ values, with missing data imputed using Perseus^29^ with default settings. Regulated proteins were classified using Student’s t-test (class I) and the Significance B test^27,28^. Raw data are provided in Supplementary Data S1.

### Adrenocorticotropic hormone (ACTH) and corticosterone (CORT) measurement

Plasma CORT and ACTH levels were measured using commercially available ELISA kits (ACTH: sensitivity < 1 pg/mL, intra- and inter-assay CV ≤ 10.3%; CORT: sensitivity < 0.2 ng/mL, intra- and inter-assay CV ≤ 10.8%; IBL International, Hamburg, Germany), according to the manufacturer’s instructions^30^. In set-up 1, hormone levels were determined approximately 1 h after AS onset, whereas in set-up 2 this was performed approximately 5 h after AS onset (see Fig. 1).

### Statistics

The data were visualized and analyzed using GraphPad Prism Software (v. 9.5.1). The Venn diagram was drawn using the online BioVenn tool (www.deepvenn.com). Results are presented as mean ± SD unless otherwise indicated.

**Fig. 3:**
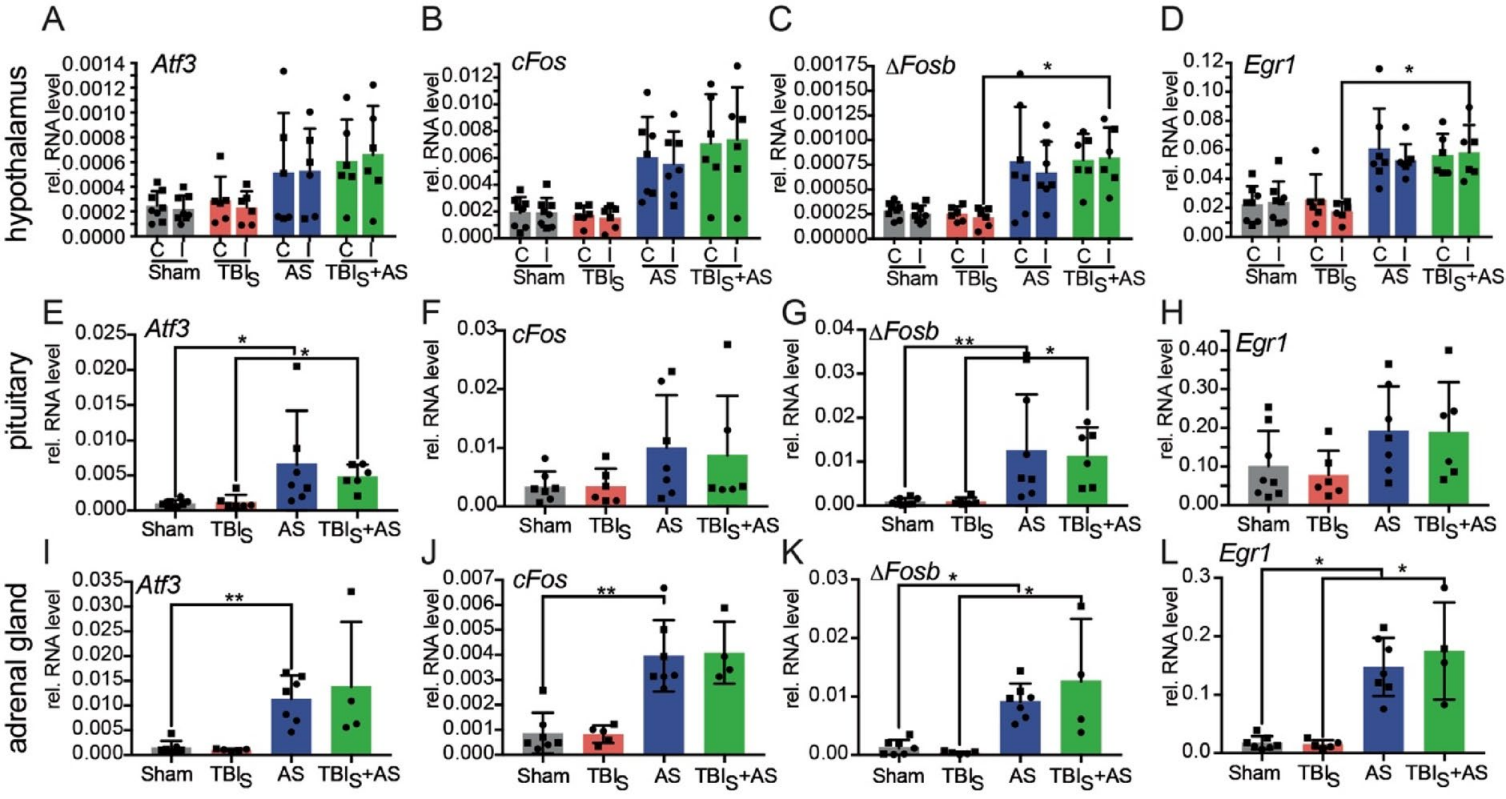
AS activated gene expression of HPA axis organs is not modulated by previous TBI exposure. (A-D) In the hypothalamus, 45 min of AS led to induction of *Atf3* (**A**) and all three IEGs (**B**-**D**). TBI before AS (TBI + AS) also allowed for this gene induction (**A**-**D**). (**E**-**H**) In the pituitary, AS alone elicited robust induction of all four genes. TBI alone performed 1 d before sacrifice expectedly did not induce the four genes. TBI did not interfere with AS-mediated induction of all four genes (TBI + AS). (I-L) A single AS exposure was sufficient to induce *Atf3* (**I**) and all three IEGs (**J**-**L**) in the adrenal gland. TBI targeted to the sensory cortex (TBI_S_) had no impact. The combination of TBI with AS induced all four genes comparably to AS alone. N-numbers are indicated by individual squares and circles in each bar. Squares represent male whereas circles represent female mice.

**Fig. 4:**
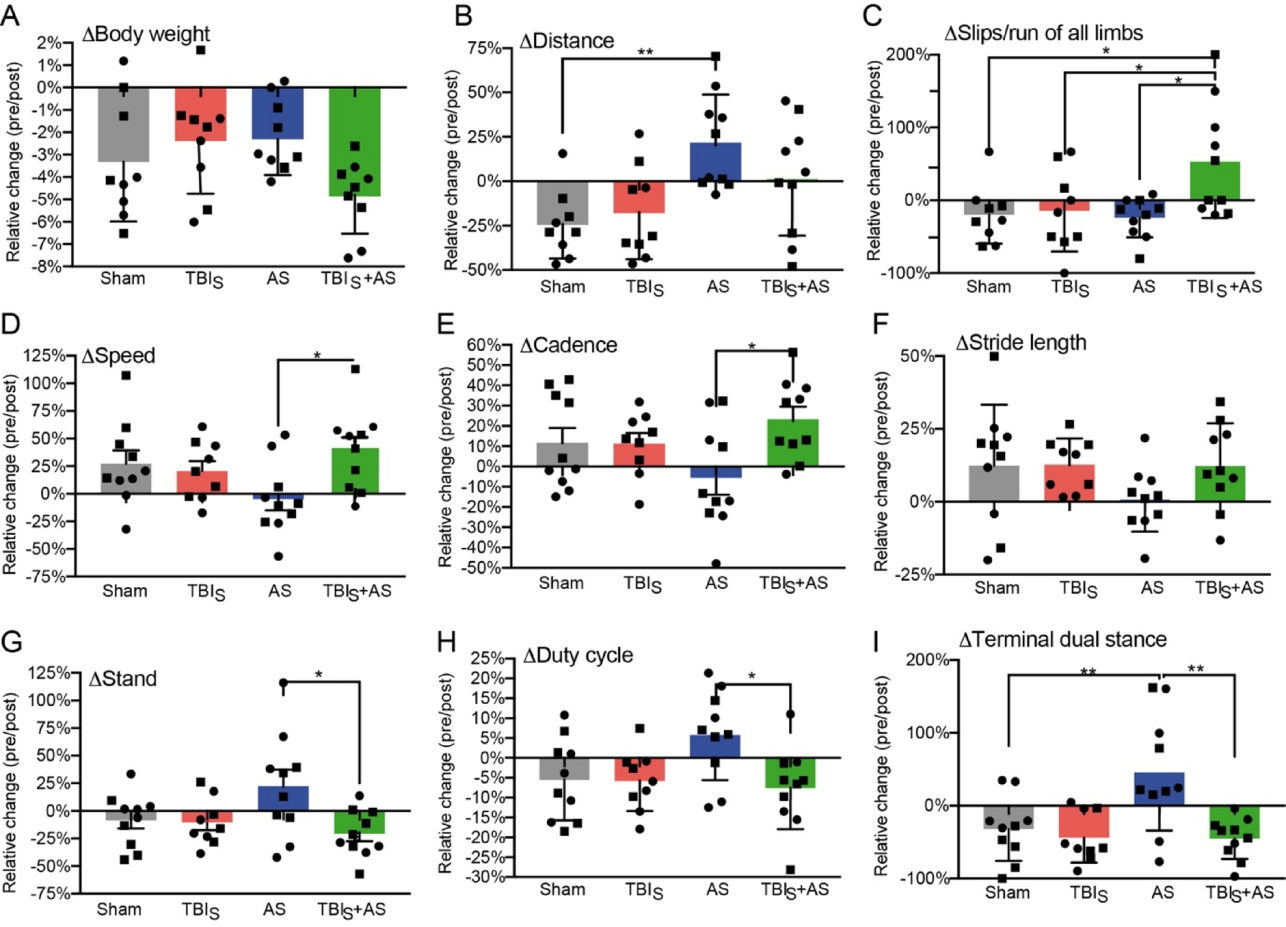
AS alters locomotor activity and gait patterns which is modulated by previous TBI challenge. Experiments were performed according to set-up 2 with 1 d (A-C) or 3 d (D-I) between TBI and AS. TBI was targeted to the sensory cortex (TBI_S_). (**A**) Body weight was reduced by 2–3% in sham and individual TBI_S_ and AS treatment. Combining TBI_S_+AS resulted in more pronounced body weight loss of approx. 5%. (**B**) In the OF, sham and TBI_S_ mice responded with reduced distance travelled compared to uninjured condition (pre/post). In contrast, single AS exposure enhanced locomotor activity. TBI exposure prior to AS prevented AS from enhancing locomotor activity (TBI_S_+AS). (**C**) In the ladder walk, sham, TBI_S_ and AS alone treated animals had almost comparable slip numbers/run as before treatment. In the combination of TBI_S_ with AS, slip numbers were enhanced indicating an interaction of both challenges. (D-I) Several gait parameters in the Catwalk system were quantified. AS alone was able to modify several gait parameters including speed (**D**), cadence (**E**), stride length (**F**), stand (**G**), duty cycle (**H**) and terminal dual stance (**I**) compared to sham and TBI alone. All six AS modulated gait parameters were reverted to similar levels of sham treated animals when TBI preceded AS. All data depict the ratio of gait parameters obtained before (pre) vs. after (post) challenge application. N-numbers are indicated by individual squares and circles in each bar. Squares represent male whereas circles represent female mice.

**Fig. 5:**
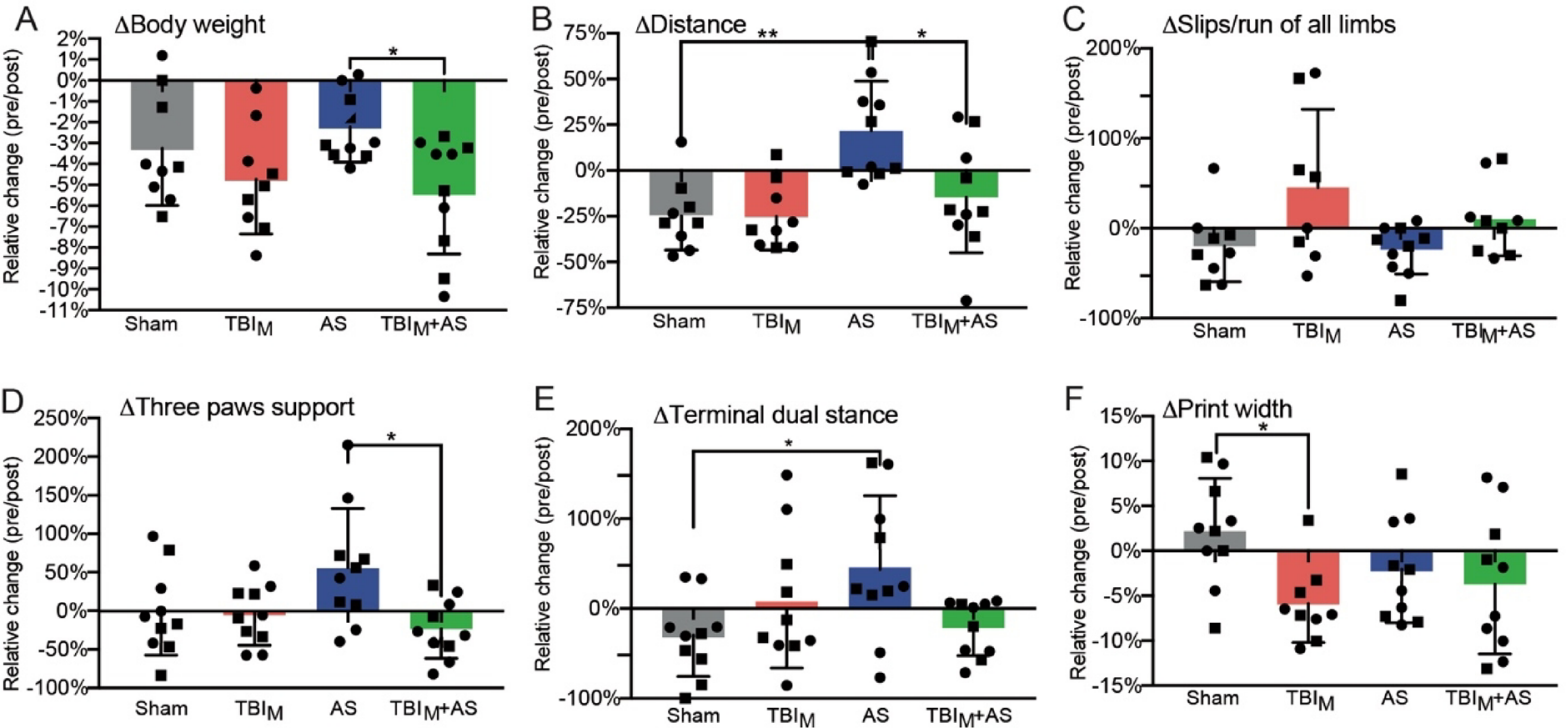
Previous TBI targeting the motor cortex reverts AS induced behavior patterns. Experiments were performed according to set-up 2 with 1 d (A-C) or 3 d (D-F) between TBI and AS. TBI was targeted to the motor cortex (TBI_M_). Note that sham and AS alone cohorts were identical between Figs. 4 and 5. (**A**) Body weight was slightly more reduced by TBI_M_ alone compared to sham and AS alone. TBI + AS had comparable weight loss as observed for TBI alone. (**B**) TBI_M_ reduced AS induced enhancement of locomotor activity in the OF in TBI_M_+AS animals. (**C**) TBI_M_ alone enhanced slip numbers/run in the ladder walk compared to sham and AS alone. However, TBI_M_ did not cause enhanced slip numbers if succeeded by AS (TBI_M_+AS). (D-E) In the Catwalk, AS alone increased the time animals spend on three paws for support (**D**) and also terminal dual stance (**E**) whereas these parameters were unaffected by TBI_M_ alone. Of note, AS did not induce these trends for gait alterations post-TBI_M_ (TBI_M_+AS; D, E). In contrast, the print width of paws was reduced by all three cohorts (AS, TBI_M_ and TBI_M_+AS) in relation to sham suggesting no obvious impact of TBI with AS for this gait parameter. All data depict the ratio of gait parameters obtained before (pre) vs. after (post) challenge application. N-numbers are indicated by individual squares and circles in each bar. Squares represent male whereas circles represent female mice.

**Fig. 6:**
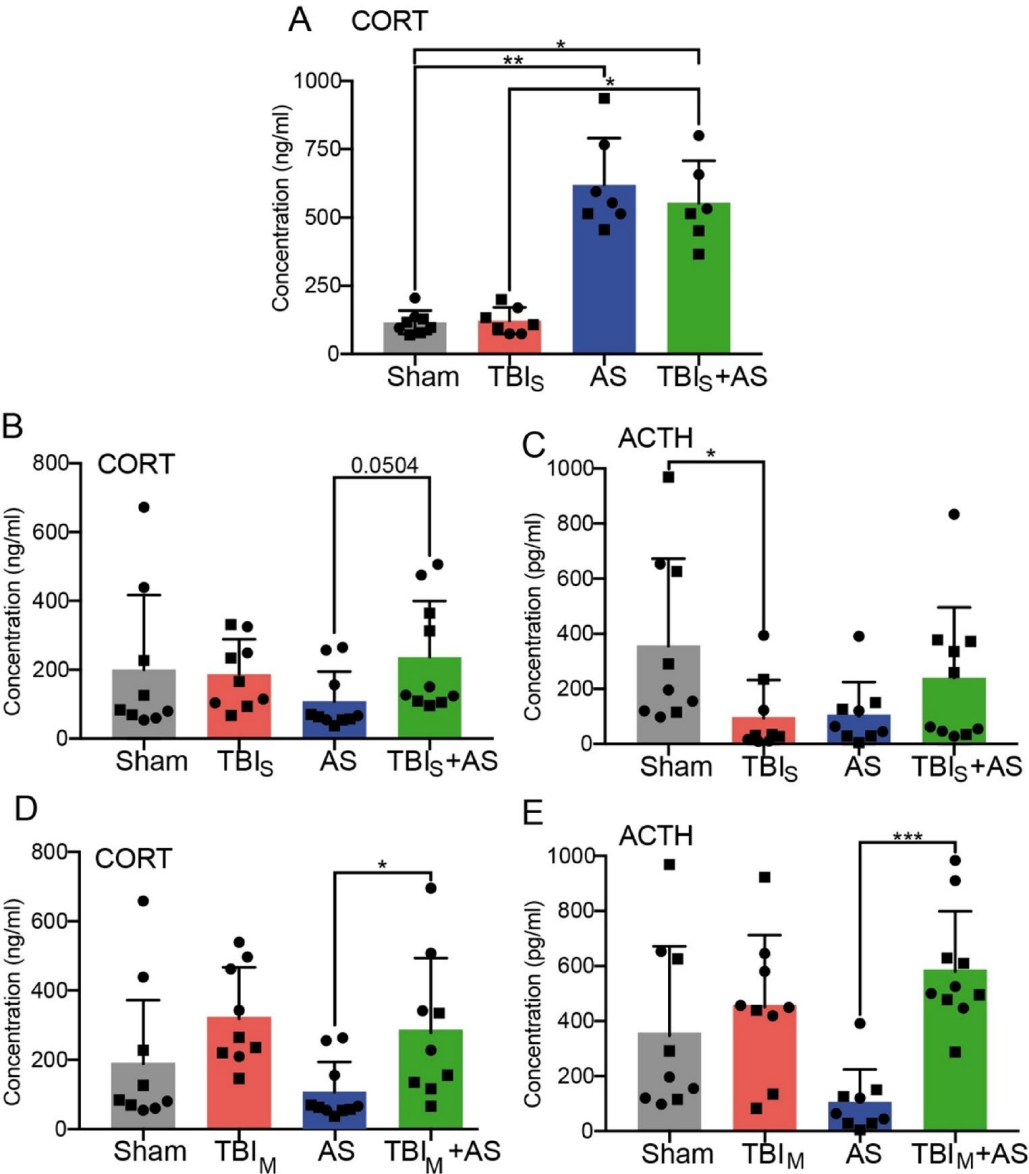
Stress hormone levels are modulated by the TBI and AS interplay. Experiments were performed according to set-up 1 (**A**) or set-up 2 (**B**-**E**) with a 1 d interval between TBI and AS. In set-up2, AS was followed by behavior tests. Note that sham and AS alone cohorts were identical in (**B**-**E**). (**A**) CORT levels in the blood were upregulated 20 min after termination of a single 45-minute AS exposure. If AS was following a TBI exposure 1 d before, CORT levels were almost the same as with AS alone. (**B**-**E**) Mice were challenged with either a sensory (TBI_S_; B, C) or a motor (TBI_M_; D, E) cortex insult. TBI_S_ alone had no impact on CORT induction (**B**) whereas ACTH release was reduced compared to sham (**C**). Of note, if AS was followed by subsequent behavior tests, both CORT (**B**) and ACTH (**C**) levels were reduced compared to sham. This is in contrast to AS without any subsequent behavior tests (**A**). If TBI_S_ occurred before AS and behavior testing, CORT (**B**) and ACTH (**C**) levels were higher compared to AS alone. TBI_M_ elevated CORT (**D**) and ACTH (**E**) levels in relation to sham. Consistent with TBI_S_+AS (B, C), AS failed to lower CORT (**D**) and ACTH (**E**) abundance when preceded by TBI_M_. N-numbers are indicated by individual squares and circles in each bar. Squares represent male whereas circles represent female mice.

**Fig. 7:**
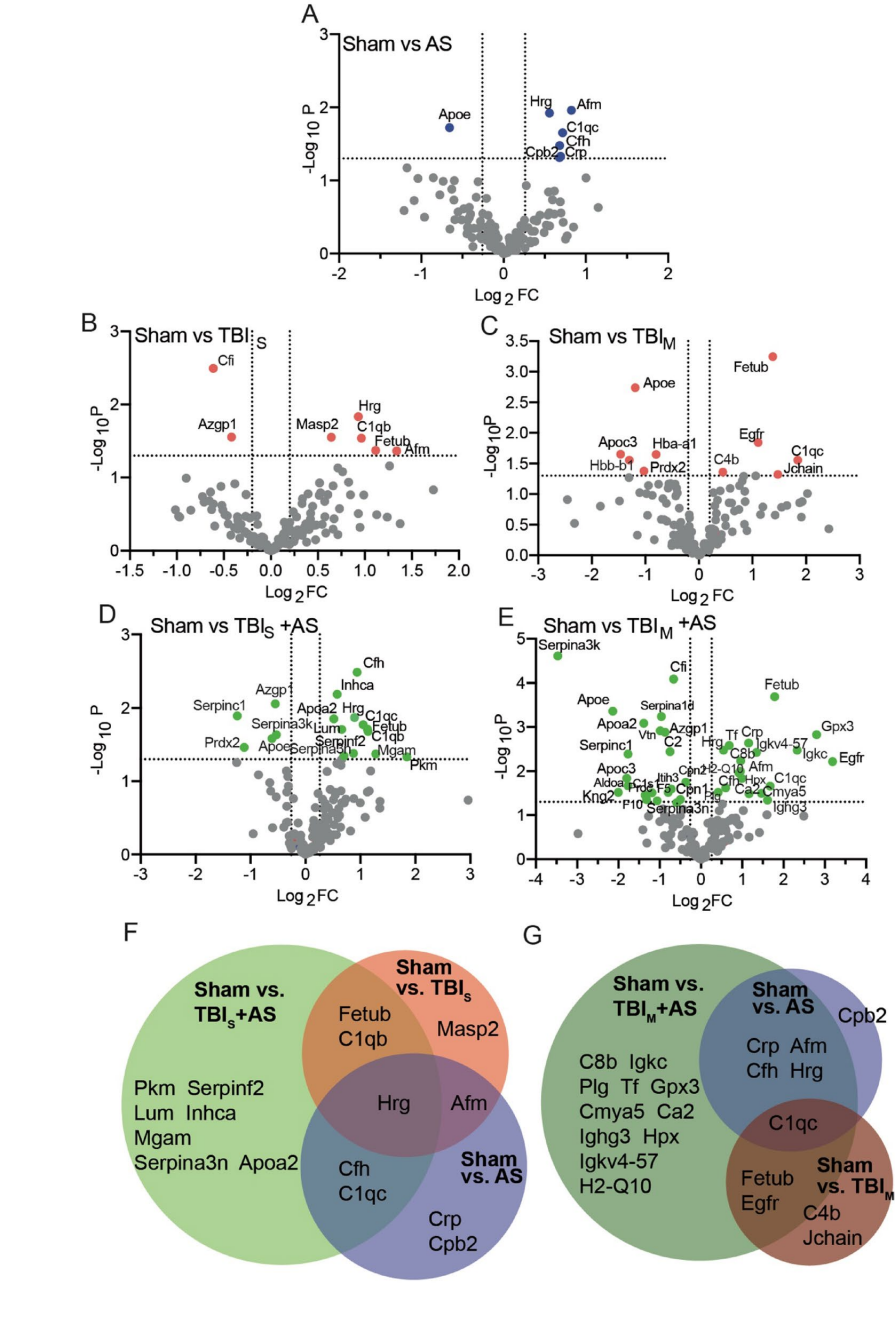
TBI and AS modulates the blood proteome. (**A**) In the AS only cohort, six proteins (Hrg, Afm, C1qc, Cfh, Crp abd Cpb2) were upregulated and one protein (Apoe) was downregulated in the blood compared to sham treated animals. (**B**, **C**) In TBI_S_ (**B**) and TBI_M_ (**C**) distinct but also shared proteins (e.g. Fetub, C1q) were upregulated when normalized to sham quantification. (**D**, **E**) Combining TBI with AS resulted in more up- and down-regulated proteins compared to AS (**A**) or TBI (**B**, **C**) alone irrespective of TBI model. (**F**, **G**) Venn diagrams of upregulated proteins in (**A**-**E**) only, highlighting proteins found in several conditions either focusing on sensory (**F**) or motor (**G**) TBI.

The number of observations for each data set is indicated in Figs. 1, 2, 3, 4, 5 and 6. Here, each circle in the bar diagrams represents one animal analyzed. Typically for each experiment in Figs. 1, 2, 3, 4, 5 and 6 the number of animals studied was between four and ten. For Fig. 7, six animals were analyzed for each of the cohorts. All information on n-numbers (including distribution of males and females) is provided in Supplementary data S2. Findings on males and females were only included descriptively and not as a factor in the statistical model.

Statistical analysis was conducted using the Kruskal-Wallis test with Dunn’s multiple comparisons, or one-way ANOVA with Tukey’s or Holm-Šídák’s multiple comparisons tests, depending on normality and homogeneity of variance. In detail, Kruskal-Wallis test followed with Dunn’s multiple comparisons test was applied in Figs. 2A-G, 3A-L, 5C and 6A-E. One-way ANOVA test followed with Holm-Šídák’s multiple comparisons test was applied in Figs. 2H and 4A-C, H and I, and Fig. 5E and F. One-way ANOVA test followed with Tukey’s multiple comparisons test was applied in Fig. 4D-G, and Fig. 5A, B, D. For a summary of all statistical results see Supplementary data S3.

Significance is denoted as * *P* < 0.05, ** *P* < 0.01, *** *P* < 0.001, **** *P* < 0.0001.

## Results

The analysis of TBI interaction with AS was performed in adult wildtype of both sexes grouped in four cohorts (sham, AS only, TBI only and TBI + AS; Fig. 1; see also Supplementary data S2). Of note, all sham mice were also subjected to anesthesia (inhaling a mixture of 4–5% sevoflurane) and pain medication (see materials & methods) thereby resulting in elevated baseline stress levels compared to completely untreated mice^20^. AS mice (blue) received a sham surgery and a 45 min restraint in a falcon tube. TBI mice (red) only received a TBI injury (Fig. 1). In the combined AS + TBI cohort (green) mice received TBI followed by AS (45 min) and 20 min of rest thereafter (Fig. 1). For qPCR, cohorts were sacrificed 20 min after completion of AS (Fig. 1A). Behavior analysis was performed in different cohorts with either 1 d or 3 d between TBI and AS exposure (Fig. 1B). Proteome analysis was performed in set-up 2. ACTH and CORT measurements were performed in both experimental set-ups (Fig. 1A and B).

### Selected gene expression responses are unaffected by TBI and AS interaction

In a first set of experiments gene expression in the cortex and septal nucleus was analyzed (see also schemes in Fig. 1C, D). Here, mice were sacrificed by carbon dioxide inhalation and hemispheres were separated in the ipsilateral (“I”), hit by the TBI impact on the sensorimotor cortex (TBI_S_) and the contralateral (“C”) hemisphere not directly hit. qPCR was performed with samples isolated approximately 1 h (45 min AS plus 20 min rest) after AS onset.

In the cortex we analyzed the TBI impact by monitoring neuroinflammatory genes (Fig. 2A-D) known to be upregulated by TBI before^23,31^. Indeed, the regeneration associated gene (RAG) *Atf3*, was upregulated by TBI only on the ipsilateral cortex (Fig. 2A). Similarly, neuroinflammatory genes (*Gfap*,* Cd68* and *Ccl2*) indicative of astrocyte or microglia activation were upregulated in mice after TBI (Fig. 2B-D). Expectedly, AS alone did not alter mRNA levels of these genes (Fig. 2A-D). When analyzing the interaction group (TBI + AS), no statistically significant difference to TBI alone was observed indicating that AS did not interfere with TBI-mediated induction of the aforementioned genes (Fig. 2A-D).

Next the impact of AS dependent induction of *Atf3* and IEGs (*cFos*,* FosB and Npas4*) was analyzed post-TBI in the septal nucleus (Fig. 2E-H), a brain region shown before to be highly susceptible to AS^20,25^. All four genes expectedly were not regulated by a TBI alone which occurred more than 1 day before (Fig. 2E-H). In contrast, AS alone upregulated all four genes in septal nuclei of both hemispheres (Fig. 2E-H) although statistically significant only for *Npas4* (Fig. 2H). The interaction of a TBI followed by AS did not show a difference in mRNA abundance of all four genes compared to AS alone (Fig. 2E-H). This suggests that TBI associated injury to neuronal cells does not interfere with AS dependent induction of these genes. Of note, both males (squares) and females (circles) behaved similarly indicating no sex specific effect (Fig. 2). However due to limited n-numbers this factor was not included in statistical testing.

In summary, in the septal nucleus AS induced RAG and IEG mRNA expression without interference by a previous TBI.

### AS activates gene expression along the HPA axis which is not modulated by previous TBI exposure

In this study we applied single 45-minute AS stimulus to analyze molecular responses in mice. So far, the impact of AS on gene signatures in HPA axis associated organs and the impact of TBI on such subsequent AS responses is not well studied.

Herein, the injury related gene *Atf3* and IEGs were analyzed for their expression levels in three HPA axis associated tissues including hypothalamus, pituitary and adrenal gland (Fig. 3). Similar to the septal nucleus in the brain (Fig. 2) single AS exposure showed a tendency toward increased expression of all four genes in both hypothalamic hemispheres, but these trends were not statistically significant (Fig. 3A-D). When AS was applied 1 d after TBI, AS still enhanced *Atf3* (Fig. 3A) and IEGs (Fig. 3B-D) to a comparable extent as with AS alone.

HPA axis activation is propagated by the hypothalamus to the pituitary and finally adrenal glands. A single AS stimulation of mice resulted in robust gene activation in both pituitary (Fig. 3E-H) and adrenal glands (Fig. 3I-L). This suggests that AS propagates a gene response down the HPA axis which was found in females and males. In contrast, 1 d after an isolated TBI (TBI alone) to the sensory cortex no gene response was observable in any of the three organs (Fig. 3) in contrast to a shorter post TBI timepoint^20^. As observed in the hypothalamus, the interaction of TBI with AS did not change *Atf3* or IEG induction in the pituitary (Fig. 3E-H) or adrenal glands (Fig. 3I-L) and similar mRNA levels as in the AS alone cohort were observed.

Taken together we show gene induction by AS-mediated HPA axis activation which is not inhibited by previous TBI experience.

### AS alters locomotor activity and gait patterns which can be modulated by previous TBI incident

In the next experiments the impact of TBI on stress related behavior was investigated (Fig. 4). For this, TBI applied to the sensory cortex (TBI_S_) was preceding the single AS stimulus by either 1 d (Fig. 4A-C) or 3 d (Fig. 4D-I).

First of all, body weight loss was measured in all four cohorts (Fig. 4A). As before^20^, sham treatment resulted in weight loss 1 d after induction. TBI individually applied did not result in more weight loss compared with sham supporting a mild TBI impact used in this study (Fig. 4A). AS alone resulted in weight loss comparable to sham and TBI alone, whereas the combination of TBI + AS showed a numerical trend toward nearly doubled weight loss compared with single AS or TBI exposure; however, this difference did not reach statistical significance (Fig. 4A).

Overall locomotor activity was assessed in the open field (OF; Fig. 4B). Here, AS alone enhanced the total distance in relation to sham as reported before^20,25^. TBI alone reduced locomotor activity similar to sham (Fig. 4B). Notably, a previous TBI reduced the AS-associated increase in locomotor activity in the OF (Fig. 4B). This suggests that TBI can influence a later AS response.

In the ladder walk, motor coordinative behavior was quantified by counting slips through the ladder rungs (Fig. 4C). AS and TBI applied individually did not cause more slips/run than sham treated animals (Fig. 4C). In contrast, when applied together in TBI + AS more slips were noted compared to single application (Fig. 4C).

Finally, the Catwalk system was applied to obtain a detailed analysis of gait parameters also allowing for detection of subtle gait changes of all four limbs (Fig. 4D-I). Of note, the time interval between TBI and subsequent AS was 3 d for this analysis.

In the six Catwalk parameters depicted here, the mild TBI_S_ applied alone did not overtly change gait parameters compared to sham (Fig. 4D-I). This was important since severe TBI models inducing e.g. limb paresis have the drawback that they impair animal locomotion so strongly that reactions to any subsequent challenge might be prevented. So far, a Catwalk analysis was not performed in acutely stressed mice. This study, shows that all six Catwalk parameters were changed in AS mice compared to sham although without reaching statistical significance (Fig. 4D-I). This included reduced speed (Fig. 4D), reduced cadence (steps/second; Fig. 4E), shorter stride length (Fig. 4F), enhanced stand time (Fig. 4G), elevated duty cycle (ratio of stand time to step cycle; Fig. 4H) and terminal dual stance (duration of ground contact for both hind paws simultaneously; Fig. 4I). Notably, for all six parameters a strong interaction with TBI was noted since AS was not able to modify all six parameters if mice experienced a TBI_S_ 3 d before (Fig. 4D-I). This was once again observed for male and female mice.

In experiments described above (Figs. 2, 3 and 4), a TBI model targeting the sensory cortex was employed (TBI_S_). This model resulted overall in a rather mild post-injury phenotype as revealed by behavior tests (Fig. 4). Thus, a different impact side, i.e. the motor cortex was also targeted by the weight-drop model to assess more severe behavior consequences after an TBI impact (labelled TBI_M_; Fig. 5). Please note that sham and AS cohorts were identical in Figs. 4 and 5.

In TBI_M_ animals, weight loss tended to be greater than in sham-treated mice, without reaching statistical significance (Fig. 5A). This is in contrast to the TBI_S_ results (Fig. 4A) where TBI_S_ treated animals had comparable weight loss compared to sham animals. The weight loss was almost identical between TBI_M_ alone and TBI_M_+AS groups (Fig. 5A) which is in opposite to the TBI_S_ results (Fig. 4A).

For the distance travelled in the OF a comparable finding between TBI_M_ and TBI_S_ was observed. In both models AS treatment post-TBI failed to enhance locomotor activity (Figs. 4B and 5B).

In the ladder walk, TBI_M_ alone resulted in more slips/run (Fig. 5C) indicating more severe TBI associated motor deficits compared to TBI_S_ alone (Fig. 4C). This TBI impact was also evident in the TBI_M_+AS group since here a tendency for more slips was observed compared to AS alone without reaching significance between both groups (Fig. 5C).

In the Catwalk, AS alone enhanced the time mice spent on all three limbs (three paws support; Fig. 5D). This parameter indicates compensatory postural adjustments to enhance stability over mobility. TBI_M_ before an AS challenge reduced the three paws support (Fig. 5D) as well as the terminal dual stance (Fig. 5E) as also seen for TBIs before (Fig. 4I). As a positive control for an individual TBI_M_ impact a reduction in print width was observed in line with previous results^22^ which was not affecting the TBI and AS interaction (Fig. 5F).

In summary, a single AS exposure impinges on gait patterns in mice which is modulated by previous TBI.

### Stress hormone levels are modulated by the TBI and AS interplay depending on experimental set-up

Above we observed that stress associated gene regulation of the HPA axis was not affected post-TBI exposure (Fig. 3). In order to analyze this further on the level of stress hormone production, ACTH and CORT levels were quantified in the blood of the four cohorts in both experimental set-up 1 and 2 (Figs. 1 and 6).

In the first set-up, mice were sacrificed approx. 1 h after AS initiation without any further behavioral testing (Figs. 1A and 6A). Here, CORT was expectedly strongly induced in the blood of AS alone treated animals (Fig. 6A). The CORT levels of AS alone treated mice were likewise achieved if TBI occurred 1 d before the AS exposure (TBI_S_+AS: Fig. 6A). Thus, TBI did not have any impact on subsequent AS-mediated CORT induction in this set-up where hormones were measured approx. 1 h after AS onset.

Next, we tested cohorts in set-up 2 (Fig. 1B) where mice after AS were subjected to further behavioral testing (OF, ladder walk, Catwalk) for approx. 5 h before sacrifice and measuring hormone levels (Fig. 6B-E). Of note, when mice after single AS experience were further challenged with extensive behavioral testing, CORT and ACTH levels were not induced anymore and were already declining (Fig. 6B-E). In fact, CORT and ACTH levels were lower than in sham treated animals. This might be indicative of the HPA axis associated negative feed-back loop where after a prolonged time of activation, CORT suppresses CRH and ACTH release from the hypothalamus and pituitary, respectively. When TBI alone was analyzed, a difference between both TBI models, TBI_S_ (Fig. 6B, C) and TBI_M_ (D, E) was noticed. In TBI targeted to the sensory cortex, CORT levels were unchanged and ACTH lower compared to sham treated mice (Fig. 6B, C). In contrast, TBI affecting the motor cortex resulted in CORT induction and slight ACTH upregulation compared to sham (Fig. 6D, E). Notably, when TBI was preceding AS (TBI + AS), higher CORT and ACTH levels were achieved compared to AS alone regardless of which TBI model was applied (Fig. 6B-E).

These results suggests that a AS dependent downregulation of CORT and ACTH levels can be inhibited post-TBI depending on the experimental time course.

### TBI and AS modulates the blood proteome

Above we demonstrated that the TBI and AS interplay modulated behavior and hormone levels regulated by single AS challenge. In order to get hold of proteins associated with this interplay we performed proteomics in the blood samples of all four cohorts in experimental set-up 2 (Figs. 1B and 7; see also Supplementary Data S1).

Mice subjected to 45 min of AS upregulated only a few proteins and down-regulated one protein (Fig. 7A). These included the complement factor C1qc and H (Cfh), the C-reactive protein (Crp) and Hrg (Histidine-rich glycoprotein; Fig. 7A).

In mice treated with TBI alone we observed more proteins to be regulated compared to AS alone irrespective of the TBI model, TBI_S_ (Fig. 7B) or TBI_M_ (Fig. 7C). Interestingly, in TBI alone we found proteins such as Hrg, Afm (Afamin), apolipoproteins (Apoe) and complement factors also regulated by AS alone (Fig. 7A-C). Several apolipoproteins were already previously described to be TBI regulated^20^. This suggests that such proteins in the blood were upregulated by such diverse exposures as AS or TBI. Furthermore, Fetub (FetuinB), a liver-secreted acute-phase glycoprotein known to protect neurons in TBI^32^, was upregulated in both TBI models (Fig. 7B, C).

In mice with an TBI and AS interaction (TBI + AS), the number of up- and downregulated proteins was higher compared to individual application in both TBI models (Fig. 7D, E) suggesting a synergistic effect. Fetub was found in both mouse cohorts of TBI_S_+AS and TBI_M_+AS supporting a reliable induction of this protein upon TBI (Fig. 7D-G). In AS alone, six proteins were upregulated (Fig. 7A, F-G). Out of these six proteins, three (Hrg, Cfh, C1qc) or four (Crp, Afm, Cfh, Hrg) were also found in TBI_S_+AS and TBI_M_+AS, respectively (Fig. 7D-G) suggesting that previous TBI did not strongly interfere with AS dependent modulation of the blood proteome. Only Cpb2 (Carboxypeptidase2) which was upregulated by AS alone was not found in any TBI + AS condition (Fig. 7F, G) suggesting that this induction was prevented post-TBI.

In summary, proteomics analysis revealed known and novel proteins regulated by TBI or AS alone but also by their interaction.

## Discussion

Herein we analyzed the consequences of prior TBI exposure on subsequent behavioral and neuroendocrine response to AS.

So far, gene expression responses altered by a TBI and AS interplay were not well investigated in the literature. To investigate AS dependent gene induction, IEGs were employed. Herein, AS elicited an IEG response in the brain (Figs. 2 and 3) in agreement with previous data^20,25^. Outside the brain, we made the finding of AS alone resulting in IEG induction in HPA axis organs including pituitary and adrenal glands (Fig. 3) in line with previous records^33^. Whether this IEG induction is consequence or cause of AS driven ACTH or CORT secretion by the pituitary and adrenal gland, respectively, is currently unknown. In any case, our findings implicate IEGs as markers for HPA axis activation.

In all tissues analyzed, TBI did not interfere with AS induced IEG induction (Figs. 2 and 3). In the brain, IEG induction is mainly found in neurons and serves as surrogate for their activation^34^. Therefore, TBI - induced one day before AS - did not prevent neurons from responding to AS with IEG induction. Since IEG induction reflects activated neurons this also indicates no interference of TBI with AS-mediated neuronal firing although direct physiological proof is missing.

Besides the brain, TBI also did not interfere with further gene activation along two HPA axis organs, pituitary and adrenal glands. This was also supported by comparable CORT levels in AS only and TBI + AS animals of set-up 1 (Fig. 6A). Overall, this implies that under experimental conditions used in this study, two parameters, IEG gene response and CORT induction, were TBI-independently triggered by AS.

In contrast to gene expression, TBI exposure had an impact on subsequent AS responses on behavior level (Figs. 4 and 5).

First of all, first observations were made that a single 45-minute lasting AS exposure resulted in modulation of gait parameters of mice. Up until now, single AS stimuli reportedly enhance locomotor and exploratory activity^20,25^. However, so far, alterations in gait patterns occurring shortly after single AS exposures were not reported in rodents. In humans, a single study reported no major AS impact on movements most likely due to insufficient stress intensity employed^35^. In rodents, we noted such tendencies for AS induced gait parameter alterations already shortly after AS completion, i.e. within approx. 3 h (Figs. 4 and 5). Interestingly, the gait changes inflicted by AS including slower walking speed, shorter step length, reduced cadence and balance deficiency (Figs. 4 and 5) resemble gait alterations described for humans with anxiety related disorders^36^. Therefore, Catwalk analysis after an AS paradigm might be suited to investigate anxiety-related movement alterations. In addition, other tests (e.g. light-dark box and elevated plus maze) should be performed to strengthen this point.

Next, we observed that such AS-inflicted behavior (gait changes, OF locomotor activity; Figs. 4 and 5) could be modulated if animals experienced TBI before. As both sensory (Fig. 4) and motor cortex (Fig. 5) directed TBI did modulate AS-associated behaviors, several cortical injury types were able to modulate stress-associated behavior. Of note, both TBI models were rather mild compared to previous TBI models^23^. However, for this study mild behavior consequences of TBI were crucial since more severe TBI models introducing massive weight loss and e.g. paresis would interfere with any meaningful subsequent behavioral challenge such as AS exposure used in this study.

Mechanistically, it is currently unclear how TBI influenced subsequent AS responses. However, we noted a correlation in the TBI + AS cohorts between gait alterations returning to sham levels (Figs. 4 and 5) and elevated levels of ACTH and CORT (Fig. 6). Thus, in the behavior cohorts (set-up 2; Fig. 1B), HPA axis-mediated CORT and ACTH induction were increased by TBI + AS in relation to AS in line with a previous report^16^. However, this was only observed in experimental conditions were AS was followed by other stressors due to additional behavior tests (Fig. 6B-E). Nevertheless, these findings might indicate a connection of HPA axis signaling with trends for gait pattern alterations associated with AS.

In addition to HPA axis activity, further modulation of an TBI and AS interplay might be provided by blood circulating proteins identified in this study (Fig. 7). Proteins identified cluster in functional units such as immune regulation (Fig. 7). For instance, complement cascade proteins including C4b, Cfh, C1qb, C1qc, C8b, Cfi and C2 and the C-reactive protein (Crp) were regulated by individual or combined TBI or AS application (Fig. 7A-E). This agrees with previous records in humans as well as animal models showing complement activation in TBI^37^ and acute stress^38^. A further inflammation associated protein is provided by Crp, an innate acute phase protein produced by the liver. Crp is an established marker in TBI diagnosis^39^ and is modulated by AS in patients^40^.

A different protein class modulated by individual or combined TBI and AS were apolipoproteins such as Apoa2, Apoe and Apoc3 identified in this study (Fig. 7). This is congruent with apolipoprotein isoforms having functions besides lipid transport and reported functions as established modulators of stress and traumatic injury as described before^41^.

Finally, Carboxypeptidase 2 (Cpb2) was induced by AS (Fig. 7). In line with this finding, carboxypeptidases were shown to be induced by restraint stress and mediate neuronal protection during AS^42^. Of note, in TBI + AS animals, Cpb2 upregulation was absent (Fig. 7) indicating loss of a *bona fide* neuroprotective factor in this condition with its associated behavior phenotypes (see above).

An important question for the interaction of TBI with AS responsiveness is the time line by which TBI still can impact on subsequent AS responses. Herein we employed 1–3 days between challenges and observed that within this time window TBI could impact on some (e.g. behavior) but not all parameters (e.g. gene induction).

In future research it would be interesting to decipher the longest time interval by which TBI still impacts on AS responses and determine a temporal limit after which a “TBI memory” is eradicated by the brain so that AS responses function physiologically.

### Limitations

This study has several limitations which should be taken into account for data interpretation:

i) The estrus cycle was not monitored in female animals which may have resulted in different hormone levels at the timepoint of behavioral testing.
ii) CO_2_ exposure induces ACTH levels. We tried to keep CO_2_ exposure to the minimum and identical for all cohorts. However, euthanasia might have had an impact on ACTH testing.
iii) We performed extensive behavioral testing (lasting approx. 4 h) before measuring HPA axis hormone levels. ACTH and CORT levels typically peak already within 15 min after stress exposure and thus prolonged stress exposure used in this study might trigger a negative feed-back on HPA axis induction.
iv) animal numbers were too low to allow for separate analysis of male and female animals.

## Supplementary Information

Below is the link to the electronic supplementary material.

Supplementary Material 1

Supplementary Material 2

Supplementary Material 3

## Data Availability

The data that support the findings of this study are available from the corresponding author upon reasonable request.
